# Effect of drinking water source on associations between gastrointestinal illness and heavy rainfall in New Jersey

**DOI:** 10.1371/journal.pone.0173794

**Published:** 2017-03-10

**Authors:** Jessie A. Gleason, Jerald A. Fagliano

**Affiliations:** Environmental and Occupational Health Surveillance Program, New Jersey Department of Health, Trenton, NJ, United States of America; National Sun Yat-sen University, TAIWAN

## Abstract

Gastrointestinal illness (GI) has been associated with heavy rainfall. Storm events and periods of heavy rainfall and runoff can result in increased microbiological contaminants in raw water. Surface water supplies are open to the environment and runoff can directly influence the presence of contaminants. A time-stratified bi-directional case-crossover study design was used to estimate associations of heavy rainfall and hospitalizations for GI. Cases of GI were identified as in-patient hospitalization with a primary diagnosis of infectious disease associated diarrhea [ICD-9 codes: specified gastrointestinal infections 001–009.9 or diarrhea 787.91] among the residents of New Jersey from 2009 to 2013 resulting in a final sample size of 47,527 cases. Two control days were selected on the same days of the week as the case day, within fixed 21-day strata. Conditional logistic regression was used to estimate odds ratios controlling for temperature and humidity. To determine potential effect modification estimates were stratified by season (warm or cold) and drinking water source (groundwater, surface water, or ‘other’ category). Stratified analyses by drinking water source and season identified positive associations of rainfall and GI hospitalizations in surface water systems during the warm season with no lag (OR = 1.12, 95% CI 1.05–1.19) and a 2-day lag (OR = 1.09, 95% CI 1.03–1.16). Positive associations in ‘Other’ water source areas (served by very small community water systems, private wells, or unknown) during the warm season with a 4-day lag were also found. However, there were no statistically significant positive associations in groundwater systems during the warm season. The results suggest that water systems with surface water sources can play an important role in preventing GI hospitalizations during and immediately following heavy rainfall. Regulators should work with water system providers to develop system specific prevention techniques to limit the impact of heavy rainfall on public health.

## Introduction

Heavy rainfall has been associated with outbreaks of waterborne diseases [1–3], and specifically with increases in gastrointestinal illness (GI) [4–6]. Storm events and periods of heavy rainfall and runoff can result in increased raw water turbidity, which has been positively associated with microbiological contaminants in raw water [5, 7]. Outbreaks of waterborne diseases have been attributed to unfiltered, insufficiently filtered, or inadequately disinfected surface waters [1, 8]. Surface water supplies, which are open to the atmosphere and to direct runoff from the land, are therefore susceptible to microbiological contamination [7]. Particles, indicated by high turbidity levels, can protect pathogens from inactivation by chemical disinfection [9, 10]. Source water turbidity as an indicator of water quality has been shown to be an explanatory factor in GI [11–16].

Unlike surface water, groundwater is thought to be naturally protected from increases in turbidity and microbial contamination from rainfall and runoff and therefore does not require disinfection plus filtration for particle removal [17]. However, national waterborne disease surveillance data show that the majority of drinking water-related outbreaks are occurring in community water systems that utilize a groundwater source [18, 19]. Non-disinfected groundwater may be vulnerable to contamination with viruses, which can increase the risk of GI among communities served by these systems [20]. Microbes can be introduced into the distribution system and without a disinfection residual there is no first line of defense [18]. A systematic review identified the need for ‘robust epidemiological studies’ to assess health risks associated with other types of water systems as well as to determine the influence of precipitation on GI [21].

Since severe weather events are predicted to occur more frequently and with greater severity in the future [22], a more thorough understanding is needed of the conditions under which heavy rainfall may impact microbiological water quality and result in increases in GI. Additionally, assessing the role of drinking water source will help to fill in identified gaps in this area of research [21, 23]. To assess our hypothesis that days of heavy rainfall are associated with increased risk of hospitalization due to GI, we employed a time-stratified bi-directional case-crossover study design. Additionally, we hypothesize risk might differ by drinking water source, so analyses were stratified by drinking water source based on residential address. Findings from this work can be used to provide guidance for interventions to prevent disease and protect drinking water sources.

## Materials and methods

### Ethical statement

This research involves human participants’ personal health information and was approved by the New Jersey Department of Health (NJDOH) Institutional Review Board (IRB) for human subjects research and later transferred to Rowan University IRB as the IRB of record for this study. Informed consent for this work was waived due to practicability and determination of minimal risk.

### Study population and GI case information

The study population comprised residents of the State of New Jersey during 2009 through 2013. Cases of GI among the population were identified as in-patient hospitalizations with a primary diagnosis of infectious disease associated diarrhea [ICD-9 codes: specified gastrointestinal infections 001–009.9 or diarrhea 787.91] in the New Jersey Hospital Discharge Data Collection System.

### Study design

We employed a time-stratified bi-directional case-crossover study design, which compares each case’s exposure to referent (control) days. Since each case serves as his or her own control, individual-level confounding factors that remain constant over a short period of time (e.g., age, race, gender, socioeconomic status) are controlled for.

The date of hospital admission served as the case day. Two control days were selected from those days on the same day of the week as the case, and that were within the same, fixed 21-day stratum as the case (Twenty-one day strata began on January 1, 2009). Further, bi-directional control sampling allows theses control days to be selected from weeks before and/or weeks after the case day [24]. Selection by week avoids any confounding effects of day of week and time-stratification into 21-day strata avoids bias from long-term temporal patterns [24–27]. Reoccurring hospitalizations for an individual within the same 21-day stratum were excluded to maintain an assumption of independence [27, 28].

### Data sources

#### Rainfall data and other weather factors

Global Surface Summary of the Day data, which includes daily measures of rainfall, temperature, and relative humidity for 26 stations in New Jersey, were obtained from the National Centers for Environmental Information [29]. Each of the 26 stations were geocoded to one of five climate regions defined by the Office of the New Jersey Climatologist [30]. Daily values of meteorological factors from each station were averaged within each climate region to minimize the impact of missing daily data at any one weather station.

#### Community water system boundaries

The New Jersey Department of Environmental Protection (NJDEP) developed a geographic information system (GIS) layer defining the boundaries of community water system (CWS) in the state [31]. Each of the 601 identified CWSs was categorized into one of two main sources of water: groundwater or surface water. This categorization was made using data reported to the U.S. Environmental Protection Agency (USEPA) and also from data collected by NJDEP [32, 33]. If there was a discrepancy in water source classification between the two sources, the USEPA classification was used. An estimated 12 percent of New Jersey residents are served by private wells or very small community water systems not mapped in the GIS layer (e.g., mobile home parks). Since water sources in these areas may pose a unique microbiological risk, these areas were designated as ‘Other’ water source.

#### Other data

Using the 2010 U.S. Census Bureau, the percentage of persons living in poverty by census tract was used to define socioeconomic status (SES) [34]. A high proportion of people living in poverty results in a low SES categorization. For use of SES as a stratifying variable in this analysis, high SES was defined as < 5% living in poverty, medium SES as 5 to 20%, and low SES as >20%.

### Exposure assessment

Each GI case was geocoded using the residential street address appearing in the hospitalization record, with 94% completed matching. Cases that could not be geocoded to residential address were excluded. Cases were spatially linked to one of the five identified climate regions in New Jersey. Each case-day and control-day was assigned meteorological exposures based on day and the assigned climate region. Each case was also spatially linked to a New Jersey CWS or ‘Other’ water source area, and to a census tract.

### Statistical analyses

Conditional logistic regression was used to estimate the relative odds of exposure to rainfall events, comparing case days to control days. Odds ratios (OR) and 95% confidence intervals (CI) were computed. Rainfall was categorized as ‘heavy rainfall’ if it was equal to or greater than the 90^th^ percentile (0.37 inches) for same day rainfall or (0.33 inches) for 3-day average rainfall among control days. Previous research has indicated that associations with precipitation and outbreaks of disease occur at or above the 90^th^ percentile [1, 10]. All models were adjusted for same day temperature and same day relative humidity. Statistical significance was determined if the 95% confidence interval did not overlap the null.

Microbiologically-associated GI hospitalizations could have latency periods that include the time from a rainfall event to exposure of an individual at the tap, the time from exposure to symptom onset, and the time from symptom onset to hospitalization. Therefore, exposure lagging of up to 14 days was explored. To explore the potential for cumulative impact of rainfall events, 3-day average rainfall was computed with 3-day, 5-day, and 7-day lags.

Stratification by season defined as warm (April through October, average temperature ≥ 50 degrees Fahrenheit) or cold (November through March) was performed to assess seasonal effect modification by season. Models were also stratified by water source: groundwater, surface water, and ‘Other’ to assess effect modification by water source. Finally, models were stratified by gender, age group (≤ 5 years, 5 < 65 years, ≥ 65 years) since the pediatric and older populations may be more susceptible to GI events, race (White, Black, Asian, American Indian/Alaska Native, and other/unknown), and census tract-based SES. Based on findings, a threshold analysis was conducted in which the bottom 50^th^ percentile was compared to each increasing 5^th^ percentile up to the 90^th^ percentile for each water source. ORs and 95% CIs were graphed to explore the shape of the dose-response relationship.

## Results

A total of 47,527 cases of hospitalization for GI were identified, and resulted in the selection of 95,054 control days. The service population of CWS across the state varies widely. Therefore, a large proportion of cases were linked with larger CWSs. One CWS served by a surface water source served approximately 11% of cases, and supports over 790,000 people (about 9% of New Jersey’s population). Six other systems each served about 5% of the cases. The ‘Other’ category consisting of areas served by very small CWS, private wells, or unknown was 15% of the study population, which compares to the current estimate of 12% of the New Jersey population served by private wells.

Cases were more often female (59.0%) than male (41.0%). A larger proportion of cases were White (67.3%) than Black (20.5%) or other race group. About 5% of cases were under age 5 years, and 43% were age 65 years or over. Approximately 18% of cases lived in census tracts categorized for this analysis as low SES. Demographic characteristics of cases differed by drinking water source (Table 1). From surface water sources, there were higher proportion of cases who were non-White, younger, and from low SES census tract.

**Table 1:** Percentage of cases by gender, race, age group, and SES demographic characteristics by water source type.

		Ground(20.3%)	Surface(64.5%)	Other(15.3%)
Gender				
	Male	19.2	65.8	15.0
	Female	21.0	63.5	15.5
Race				
	White	23.6	58.9	17.4
	Black	13.0	76.1	10.9
	Asian	9.1	80.5	10.3
	AI/AN	18.7	72.4	8.9
	Other/Unknown	14.5	74.3	11.2
Age group				
	<5 years	16.2	72.5	11.2
	≥5 and <65 years	18.2	66.6	15.3
	≥ 65 years	23.3	60.9	15.8
SES				
	High SES	21.8	59.7	18.5
	Moderate SES	22.1	64.6	13.3
	Low SES	12.3	74.2	13.5

Adjusted ORs and 95% CIs of GI hospitalization and heavy rainfall are presented for various lag periods for the full year and by season in Table 2. Effect estimates up to lag 14 were estimated but only findings up to lag 7 are presented, since there were no significant associations seen with lags beyond that point.

**Table 2:** Adjusted odds ratios (OR) and 95% confidence intervals (CI) of association with 90^th^ percentile precipitation and hospitalizations for gastrointestinal illness by season.

	Full YearN = 142,581		Warm SeasonN = 81,125		Cold SeasonN = 61,456	
	OR	95% CI	OR	95% CI	OR	95% CI
No lag	1.02	0.98–1.05	**1.10**	1.04–1.15*	**0.91**	0.86–0.97*
1-day lag	0.97	0.93–1.01	1.04	0.99–1.09	**0.87**	0.82–0.92*
2-day lag	1.02	0.98–1.06	**1.09**	1.04–1.14*	**0.92**	0.87–0.98*
3-day lag	**0.96**	0.92–0.99*	1.03	0.98–1.08	**0.85**	0.80–0.90*
4-day lag	1.03	0.99–1.07	**1.07**	1.02–1.12*	0.96	0.91–1.02
5-day lag	1.02	0.98–1.06	1.05	1.00–1.10	0.99	0.93–1.05
6-day lag	1.00	0.96–1.03	1.02	0.97–1.06	0.96	0.91–1.02
7-day lag	0.99	0.96–1.03	0.99	0.94–1.04	0.98	0.92–1.04
3-day avg (no lag)	0.96	0.92–0.99	1.03	0.98–1.08	**0.86**	0.81–0.92*
3-day avg (3-d lag)	0.99	0.96–1.03	1.03	0.98–1.08	**0.94**	0.88–0.99*
3-day avg (5-d lag)	0.99	0.96–1.03	0.97	0.92–1.02	1.03	0.97–1.09
3-day avg (7-d lag)	**1.04**	1.01–1.08*	1.02	0.97–1.07	1.06	1.00–1.12

ORs adjusted for temperature and humidity; Warm season (April–October), Cold Season (November-March); N is number of case and control days.

*Statistically significant (p-value < 0.05).

For the full year, there was an inverse association of GI with rainfall with a 3-day lag (OR = 0.96, 95% CI 0.92–0.99) and a positive association of GI with 3-day average rainfall with a 7-day lag (OR = 1.04, 95% CI 1.01–1.08). During the warm season, positive associations of GI hospitalization and heavy rainfall were seen with no lag (OR = 1.10, 95% CI 1.04–1.15), a 2-day lag (OR = 1.09, 95% CI 1.04–1.14), and a 4-day lag (OR = 1.07, 95% CI 1.02–1.12). During the cold season there were statistically significant inverse associations for same-day lag through 3-day lag, and for 3-day averages with no lag and a 3-day lag.

Table 3 presents adjusted ORs and 95% CIs for GI hospitalization and rainfall stratified by drinking water source as well as season. For groundwater systems, there were no statistically significant associations between rainfall and GI during the warm season. During the cold season, there were inverse associations with a 3-day average rainfall with no lag (OR = 0.82, 95% CI 0.70–0.96) with a 7-day lag (OR = 0.86, 95% CI 0.75–1.00).

**Table 3:** Adjusted odds ratios (OR) and 95% confidence intervals (CI) of association with 90^th^ percentile precipitation and hospitalizations for gastrointestinal illness by season and water source.

	Groundwater				Surface water				Other			
	WarmN = 16284		ColdN = 12584		WarmN = 52254		ColdN = 39658		WarmN = 12587		ColdN = 9214	
	OR	95% CI	OR	95% CI	OR	95% CI	OR	95% CI	OR	95% CI	OR	95% CI
No lag	1.05	0.93–1.18	0.93	0.80–1.08	**1.12**	1.05–1.19*	**0.90**	0.83–0.97*	1.06	0.93–1.20	0.98	0.83–1.15
1-day lag	1.02	0.91–1.14	0.88	0.76–1.02	1.05	1.00–1.11	**0.85**	0.79–0.91*	1.01	0.89–1.14	0.94	0.80–1.10
2-day lag	1.08	0.96–1.20	0.98	0.85–1.12	**1.09**	1.03–1.16*	**0.89**	0.83–0.96*	1.07	0.95–1.20	1.01	0.86–1.19
3-day lag	1.00	0.90–1.12	0.89	0.76–1.03	1.02	0.96–1.07	**0.83**	0.77–0.89*	1.11	0.99–1.25	0.89	0.75–1.04
4-day lag	1.06	0.95–1.19	0.97	0.84–1.12	1.06	1.00–1.12	0.95	0.88–1.02	**1.16**	1.03–1.31*	1.02	0.87–1.19
5-day lag	1.06	0.95–1.19	1.05	0.92–1.21	1.03	0.98–1.09	0.98	0.91–1.05	1.10	0.97–1.24	0.97	0.83–1.13
6-day lag	1.01	0.90–1.13	1.05	0.92–1.21	1.02	0.96–1.08	0.95	0.88–1.02	1.02	0.91–1.15	0.89	0.76–1.06
7-day lag	1.00	0.90–1.12	**0.86**	0.75–1.00*	0.97	0.92–1.03	1.01	0.94–1.09	1.05	0.93–1.19	1.01	0.86–1.19
3-day avg (no lag)	1.00	0.90–1.13	**0.82**	0.70–0.96*	1.03	0.97–1.10	**0.86**	0.80–0.93*	1.04	0.92–1.17	0.91	0.77–1.08
3-day avg (3-d lag)	0.99	0.88–1.11	1.02	0.88–1.19	1.03	0.97–1.09	**0.91**	0.85–0.98*	1.06	0.94–1.20	0.98	0.83–1.15
3-day avg (5-d lag)	0.98	0.87–1.10	1.10	0.96–1.28	0.97	0.91–1.02	0.98	0.92–1.05	0.97	0.86–1.09	**1.19**	1.02–1.39*
3-day avg (7-d lag)	1.01	0.91–1.13	1.02	0.87–1.18	1.01	0.95–1.07	1.07	0.99–1.15	1.07	0.95–1.21	1.07	0.90–1.26

ORs adjusted for temperature and humidity; Warm season (April–October), Cold Season (November-March); N is number of case and control days.

*Statistically significant (p-value < 0.05).

For surface water sources, GI was positively, statistically significantly associated with heavy rainfall during the warm season on the same day and with a 2-day lag (OR = 1.12, 95% CI 1.05–1.19 and OR = 1.09, 95% CI 1.03–1.16, respectively). During the cold season, associations for surface water sources were inversely, statistically significantly associated with same-day through 3-day lag periods as well as with a 3-day average precipitation with no lag and with a 3-day lag. For ‘Other’ water sources, there was a statistically significant positive association with a 4-day lag period (OR = 1.16, 95% CI 1.03–1.31) during the warm season, and statistically significant association with a 3-day average with a 5-day lag (OR = 1.19, 95% CI 1.02–1.39) during the cold season.

Adjusted ORs and 95% CI of associations by age group, White and Black race, and SES categories for surface water systems during the warm season are presented in S1 Table. When stratified by age group, the association between GI and heavy rainfall events was stronger among children under the age of 5 years (for example, OR = 1.41, 95% CI 1.08–1.85 for no lag) than in the other age groups. White or Black race did not appear to modify the association, while associations were stronger among individuals residing in lower SES census tracts (for example, OR = 1.20, 95% CI 1.06–1.37 for no lag).

**Fig 1:**
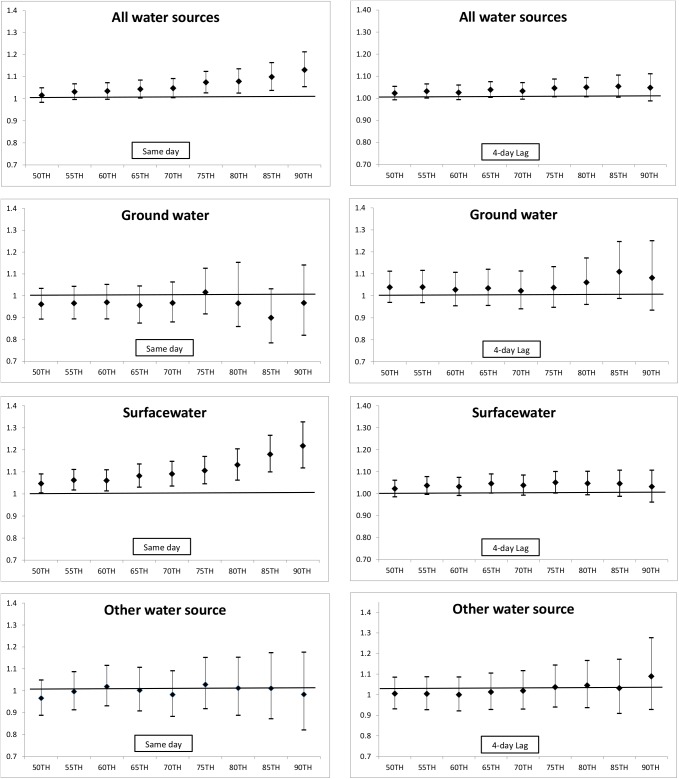
Threshold analysis of odds ratios and 95% confidence intervals of 50^th^ percentile compared to each increasing 5^th^ percentile of precipitation overall and for groundwater, surface water, and ‘Other’ water source for same day and 4-day lag precipitation during the warm season.

Threshold analysis compared the bottom 50^th^ percentile to each increasing 5^th^ percentile of rainfall levels during the warm season for both same day and 4-day lag for all water sources combined and for each water source type (Fig 1). The analysis indicates an increase in same day effect estimates of associations between GI hospitalization and rainfall with increasing rainfall amounts overall, particularly when restricted to surface water systems. The effect overall appears to be driven by associations in surface water sources. Same day (no lag) comparing the 90^th^ percentile to the 50^th^ percentile (0 inches of rainfall) produced an increased effect (OR = 1.22, 95% CI 1.12–1.33) compared to 90^th^ percentile to all other rainfall days presented in Table 3 (OR = 1.12, 95% CI 1.05–1.19). These same effects were not found when examining a 4-day lag. By removing other rainfall exposures between the 50^th^ percentile and the 90^th^ percentile are likely to have strengthen the association. An increase in effect estimate for GI hospitalization with increasing rainfall may also be indicated in ‘Other’ water source type for a 4-day lag, although no associations are statistically significant. There were no statistically significant associations for groundwater at any percentile for either same day or 4-day lag

## Discussion

Gastrointestinal illness (GI) attributable to drinking water presents a large nationwide burden each year, with estimates of 4.3 to 11.7 million cases per year [35] and 16.4 million cases per year [36]. Two reviews, one focusing on studies of precipitation and disease [23] and another on methods used to attribute GI to drinking water [21] both identified the importance of future research to assess the intersection of disease, rainfall, and drinking water factors. Our statewide study of over 47,000 hospitalizations for GI utilized a time-stratified, bi-directional case-crossover study design. Stratification by CWS water source identified positive associations of rainfall and GI hospitalizations in surface water systems during the warm season with no lag and a 2-day lag, no positive associations in groundwater systems, and positive associations in ‘Other’ water source areas (served by very small CWS, private wells, or unknown) during the warm season with a 4-day lag.

Studies have shown mixed results regarding associations of precipitation and waterborne disease. A review of studies of extreme precipitation and drinking water-related waterborne infections identified 11 studies with positive associations, six with no associations, and three with mixed results [23]. Studies in developed countries include an analysis of camplybacteriosis cases (n = 1,477) and precipitation in a 1994–2007 time-series study in Philadelphia, which found no association [37], and a time-series study of GI (n = 17,357) and rainfall in Wisconsin, 2002 to 2007 which found a statistically significant 11% increased risk [4]. The review by Guzman Herrador et al. (2015) acknowledged the limited number of studies of weather events and disease that took into account the type of water treatment, water source, or water supply.

There is evidence that indicators of microbiological drinking water quality parameters are associated with increased waterborne disease risk, and that risk may be modified by source water type. Excess risk of emergency department visits for diarrhea and source water turbidity in New York City peaked at a 6-day lag [13] and a study in Philadelphia among both the pediatric population [38] and elderly population [15] found small increases in turbidity are associated with risk of diarrheal disease. A cohort study in Vancouver, Canada found a positive association with physician visits (n = 1,353) for intestinal illness among water systems mostly served by surface water, however the same analysis examining hospitalizations of intestinal illness did not find the same positive association [38]. A cross-sectional survey in Quebec, Canada found associations with precipitation and GI with further analyses providing evidence that water source plays an important role on the risk of GI. [39].

In our study, drinking water source modified associations of rainfall and illness in which positive associations were limited to surface water and ‘Other’ sources of drinking water, but not groundwater. Threshold analyses presented in Fig 1 indicate increasing risk of hospitalizations for GI with increasing rainfall among surface water systems on the day of the rain event. These same trends were not seen in a 4-day lag and were limited to surface water sources only. This analysis strengthens the findings that associations among surface water systems are acute and increase with increasing rainfall. Teschke et al. (2010) found an increased risk of physician visits for intestinal infectious disease with two-week accumulated precipitation served by surface water supplies especially for no lag and 10-day lag but this did not remain statistically significant after adjustment for other variables [40]. Ueijo et al. (2014) found that more precipitation during the summer/fall season increased childhood GI in municipalities with untreated municipal water while GI in municipalities served by treated municipal water and private wells were not influenced by hydrologic events [41].

We hypothesized associations to be lagged due to environmental transport, disease latency, and health-seeking behaviors. Curriero et al. (2001) found a 51% increase of waterborne disease outbreaks with a 2-month lag and a time-series analysis among two Inuit communities in Canada from 2005–2008 found significantly positive associations of infectious gastrointestinal illness with high 2-week and 4-week lagged water volume input (rainfall plus snowmelt) [1, 5].

Our observed associations of little to no lag in surface water does not account for the lag time that would be associated with a hypothesized delay for a mechanism of event to exposure to disease. It is possible that there are vulnerabilities in some surface water systems which could drive these acute positive associations following rainfall, including open-air reservoirs and water storage tank susceptibilities, but future research is required to investigate this. A large time-series analysis in Wisconsin found positive associations with rainfall and GI with a 4-day lag [4] which does align with our findings when models were stratified by drinking water served by ‘Other’ water source. Additionally, comparability across studies is limited due to differences in study design, use of health outcome measures (e.g. physician visit, nurse calls, hospitalization admission), and difference in lag windows (days, weeks, months) which may affect both lagging results and overall conclusions.

Stratification by season was necessary to detect associations with rainfall and hospitalization with GI [13, 41]. Seasonal effect modification may be explained by behavioral changes such as less recreational swimming during the cold season or differences in bacterial or viral etiologies of GI during each season [41]. Interestingly, inverse associations were found during the cold season. Heavy precipitation in the cold months may frequently fall as snow, but precipitation data in this study included both rain and the rain-equivalent of snow. Impacts on water bodies may be variable depending on the timing of thaw or melting periods after snowfalls. Cold temperatures and snow- or ice-covered roads may decrease access to health care. Additionally, a patient’s choice of medical contact (in-person visits versus phone calls) appears to depend on weather conditions [42].

Our study found that children under 5 years of age were at an increased relative risk of GI hospitalizations following heavy rainfall events than other age groups, and some evidence of increased relative risk among those residing in census tracts in the lowest SES category in comparison to higher categories. Race did not modify effect estimates. Teschke et al. (2010) found an elevated odds ratio for hospitalizations for intestinal illness among children aged 1 to 4 (OR = 6.54, 95% CI 3.64, 11.8) and also individuals in the lowest two household income quintiles at an increased risk of intestinal infections (OR = 1.18, 95% CI 1.03, 1.30 and OR = 1.19, 95% CI 1.07, 1.32 respectively) [40]. Our threshold analysis indicated an increasing effect with increasing percentiles of precipitation among water systems with surface water source, but not with groundwater or ‘Other’ source.

Non-differential exposure misclassification may have introduced bias towards the null. Meteorological data were averaged from various stations over each of the five climate regions. This allowed for complete meteorological information but daily assigned rainfall exposure may not accurately reflect the actual conditions that may have impacted water quality where a particular case resides. Exposure assignment was based on residential address, but actual exposure to drinking water on any given day may have occurred elsewhere such as at work or school. Assignment of source water may have been inaccurate since some water systems, especially larger ones, are particularly complex, often utilizing both surface and groundwater in different parts of the service area and at different times throughout the year. More refined understanding of water distribution through time within complex CWSs would be needed to better classify the drinking water source for each case. The ‘Other’ category is not a homogeneous grouping regarding water quality, vulnerability to heavy precipitation, or source water type.

Although the U.S. government is responsible for implementing the Safe Drinking Water Act, each state has the authority to create more stringent and/or additional standards or rules for water systems in their jurisdiction. For example, all CWS in New Jersey are required to maintain a detectable level of disinfectant regardless of whether source water comes from a groundwater or surface water source, unlike the non-disinfected municipal water systems studied by Ueijo et al. (2014). We believe it is important to understand the role drinking water plays in endemic rates of GI especially as weather factors and events are predicted to have larger impacts in the future. However, the role of water systems in associations of precipitation and disease will likely vary based on the drinking water regulations and source waters particular to each place. Therefore, more studies in more geographic areas will be needed to understand the specific and unique vulnerabilities of water systems with varying source characteristics and regulatory environments.

Findings from this project have identified needs for further research. Additionally, more work has to be done to identify surface water system vulnerabilities. This may allow us to pinpoint the driving factors or identify water systems that may be driving observed positive associations. Future research in New Jersey may also include performing time-series analyses or analyses restricted to one large CWS served by a surface water source. This would allow a more detailed exposure assessment and may allow the linkage of rainfall, snowfall, melt events, and other possible modulating water quality parameters to GI.

## Conclusions

Water systems with surface water sources can play an important role in preventing GI hospitalizations during and immediately following heavy rainfall. Although more research is required to determine factors that may explain the mechanism of risk or to explain a mechanism unrelated to drinking water, surface water systems may consider additional protection measures during heavy rainfall events such as source water protection. Regulators should work with water system providers to develop system specific prevention techniques to limit the impact of heavy rainfall on public health.

## Supporting information

S1 Table Adjusted odds ratios (OR) and 95% confidence intervals (CI) of association with 90^th^ percentile precipitation and hospitalizations for gastrointestinal illness by age group, white/black race, and SES category in surface water sources during the warm season. (DOCX)
