# Supplementary material for: Effect of drinking water source on associations between gastrointestinal illness and heavy rainfall in New Jersey
Source: PLoS One. 2017 Mar 10;12(3):e0173794. doi: 10.1371/journal.pone.0173794 (PMC5345866; doi:10.1371/journal.pone.0173794)
Supplement: S1 Table — (DOCX) [file pone.0173794.s001.docx]

|  | Age < 5 years | | Age 5 to 65 years | | Age 65 years and older | | White | | Black | | Low SES  (High Poverty) | | Moderate SES | | High SES | |
| --- | --- | --- | --- | --- | --- | --- | --- | --- | --- | --- | --- | --- | --- | --- | --- | --- |
|  | N=2,720 | | N=28,897 | | N=20,637 | | N=31,841 | | N=12,738 | | N=11,091 | | N=23,306 | | N=17,857 | |
|  | OR | 95% CI | OR | 95% CI | OR | 95% CI | OR | 95% CI | OR | 95% CI | OR | 95% CI | OR | 95% CI | OR | 95% CI |
| No lag | **1.41** | **1.08-1.85** | **1.10** | **1.02-1.19** | **1.11** | **1.01-1.22** | **1.11** | **1.03-1.20** | 1.12 | 0.99-1.26 | **1.20** | **1.06-1.37** | 1.09 | 1.00-1.19 | 1.10 | 0.99-1.21 |
| 1-day lag | 1.04 | 0.81-1.33 | 1.04 | 0.97-1.12 | 1.07 | 0.98-1.17 | 1.05 | 0.97-1.13 | 1.04 | 0.93-1.16 | 1.05 | 0.93-1.18 | 1.02 | 0.94-1.11 | **1.10** | **1.00-1.21** |
| 2-day lag | **1.29** | **1.01-1.64** | **1.09** | **1.01-1.18** | 1.07 | 0.98-1.17 | **1.11** | **1.03-1.19** | 1.02 | 0.91-1.13 | **1.17** | **1.04-1.32** | 1.04 | 0.96-1.14 | **1.11** | **1.01-1.22** |
| 3-day lag | 1.06 | 0.83-1.37 | 1.05 | 0.97-1.13 | 0.96 | 0.88-1.05 | 0.98 | 0.91-1.05 | 1.09 | 0.97-1.22 | 1.07 | 0.95-1.21 | 1.04 | 0.96-1.13 | 0.95 | 0.86-1.04 |
| 4-day lag | 1.05 | 0.82-1.34 | 1.05 | 0.97-1.13 | 1.07 | 0.98-1.17 | 1.03 | 0.96-1.11 | **1.12** | **1.00-1.25** | 1.05 | 0.93-1.18 | 1.08 | 0.99-1.18 | 1.03 | 0.93-1.14 |
| 5-day lag | 0.98 | 0.77-1.24 | 1.03 | 0.95-1.11 | 1.05 | 0.96-1.14 | 1.02 | 0.95-1.09 | 1.08 | 0.97-1.21 | 1.04 | 0.93-1.17 | 1.03 | 0.95-1.12 | 1.02 | 0.93-1.13 |
| 6-day lag | 1.03 | 0.81-1.31 | 1.01 | 0.93-1.08 | 1.03 | 0.94-1.13 | 1.02 | 0.95-1.10 | 1.09 | 0.97-1.21 | 1.08 | 0.96-1.22 | 1.04 | 0.96-1.13 | 0.95 | 0.86-1.05 |
| 7-day lag | 0.98 | 0.76-1.27 | 1.00 | 0.93-1.08 | 0.93 | 0.85-1.02 | 0.97 | 0.90-1.05 | 0.95 | 0.84-1.06 | 0.95 | 0.84-1.07 | 1.01 | 0.93-1.10 | 0.94 | 0.85-1.03 |

**S1 Table. Adjusted Odds Ratios (OR) and 95% Confidence Intervals (CI) of Association with 90^th^ Percentile Precipitation and Hospitalizations for Gastrointestinal Illness by Age Group, White/Black race, and SES Category in Surface Water Sources during the Warm Season.**

ORs adjusted for temperature and humidity; Warm season (April-October), Cold Season (November-March); N is number of case and control days

*Statistically significant (p-value < 0.05)
